# Adverse effects of FDA-approved RNA therapeutics: a cross-platform analysis of RNA design, delivery systems, and safety profiles

**DOI:** 10.3389/fphar.2026.1935318

**Published:** 2026-10-19

**Authors:** Vassiliki Stamatopoulou, Adamantia Kouvela, Konstantina Ahtypi, Eirini Douka, Christina Kominatou, Artemis Meimaroglou, Konstantina Spyropoulou, Sofia Taxari, Sotiria Theodoridou, Panagiotis Plotas

**Affiliations:** 1 Department of Biochemistry, School of Medicine, University of Patras, Patras, Greece; 2 Postgraduate Program in Health Education, University of Patras, Patras, Greece; 3 Laboratory of Primary Health Care, School of Health Rehabilitation Sciences, University of Patras, Patras, Greece

**Keywords:** adverse effects, ASOs, mRNA, RNA drugs, siRNA

## Abstract

RNA therapeutics have emerged as a promising and effective tool to treat a wide range of genetic, metabolic, infectious, and neurodegenerative diseases by directly modulating the gene expression or directing transient protein production. Currently, more than 20 RNA-based drugs have been approved by the FDA, and due to their recent introduction into the clinical practice, long-term safety data remain limited and comparisons of adverse effects across platforms are lacking. This review focuses on the three major RNA therapeutic platforms, including antisense oligonucleotides (ASOs), small interfering RNA (siRNA) and messenger RNA (mRNA) and intends to provide a cross-platform analysis of their adverse effects. We searched studies published up to June 2026 in PubMed, Scopus, Web of Science, and Google Scholar using terms related to RNA therapeutics, individual drug names, safety and toxicity outcomes. Clinical trials, extension studies, pharmacovigilance reports, regulatory documents, and prescribing information were considered for each FDA-approved ASO, siRNA and mRNA agent. The observed safety profiles are associated with an interaction between RNA modality and additional determinants, including chemical modification, delivery system, route of administration, biodistribution, target tissue, and clinical context. Innate immune activation represents a potential shared mechanism, whereas sequence-dependent off-target effects, delivery-associated reactions, administration-related complications, and organ-specific toxicities differ in relevance and mechanism among ASO, siRNA, and mRNA therapeutics. Although most reported adverse effects are mild to moderate, important research gaps regarding long-term safety, pediatric use and rare adverse effects still exist, underscoring the need for further optimization of RNA drug design and delivery technologies, as well as continued pharmacovigilance.

## 1 Introduction

RNA therapeutics is considered one of the most important and rapidly evolving fields in modern biomedicine, fundamentally reshaping contemporary approaches to disease prevention, management and treatment at the genetic and molecular level. They use RNA molecules and RNA targeting mechanisms to regulate gene expression either at the transcriptional or translational level (Zhu et al., 2022)**.** Since the late 1990s, when the first RNA-based drug was approved using antisense oligonucleotide technology, RNA therapeutics have been rapidly developed, offering a new perspective in personalized medicine (Bajan and Hutvagner, 2020; Makkar, 2025). Their growing significance arises from unique advantages, including their high target specificity and their ability to modulate previously undruggable targets (Saw and Song, 2024). Moreover, RNA therapeutics have the privilege to be developed within accelerated timelines and minimal cost compared to conventional therapeutics (Jackson et al., 2020), while their remarkable versatility across diverse disease settings, contribute further to the advancement of personalized medicine (Qin et al., 2022).

RNA therapeutics currently comprise a broad range of platforms, including antisense oligonucleotides (ASOs), small interfering RNAs (siRNAs), modified messenger RNAs (mRNAs), microRNAs, aptamers, and RNA-guided editing tools, such as CRISPR–Cas systems (Makkar, 2025; Singh et al., 2026). Both the approved drugs and investigational agents have demonstrated substantial therapeutic potential in the treatment of a wide range of genetic disorders, autoimmune diseases, cancer, infectious diseases, and neurological conditions (Deyhimfar et al., 2024; Di Francesco et al., 2024; Boti et al., 2025). Up to date, more than twenty RNA-related therapeutics have been approved by the US Food and Drug Administration (FDA), reflecting the rapid and successful transition from experimental platforms to clinical practice and the advancement and expansion of the field (Singh et al., 2026). The present review focuses specifically on the 18 FDA-approved ASO, siRNA, and mRNA therapeutics that meet the predefined scope described in the Methods (Table 1).

**TABLE 1:** FDA-approved RNA therapeutics included in this review: Approval year, indication, delivery, administration, and reported safety profiles.

Drug	RNA platform	FDA approval (year)	Indication	Target	Delivery system	Administration route	Common adverse effects	Rare adverse effects	Organ toxicity
*Inclisiran*	siRNA	2021	Hypercholesterolemia	PCSK9	GalNAc conjugated	SC	Injection-site reactions	Hypersensitivity	No significant hepatotoxicity or nephrotoxicity
*Lumasiran*	siRNA	2020	PH1	HAO1	GalNAc conjugated	SC	Injection-site reactions, abdominal pain, headache	Hypersensitivity	No significant hepatotoxicity, nephrotoxicity or long-term systemic toxicity
*Nedosiran*	siRNA	2023	All PH subtypes	LDHA	GalNAc conjugated	SC	Injection-site reactions, as well as headache and nausea	Tachycardia	No significant hepatotoxicity
*Patisiran*	siRNA	2018	hATTR	TTR	LNP	IV	Infusion-related reactions, reduced serum vitamin A levels, URI, nasopharyngitis, influenza-like illness, peripheral edema, and fatigue	No specific rare event consistently identified in the reviewed sources	No significant hepatotoxicity, nephrotoxicity or immunogenicity
*Vutrisiran*	siRNA	2022	hATTR	TTR	GalNAc conjugated	SC	Arthralgia, pain in extremities, dyspnea, falls, peripheral edema, reduced serum vitamin A levels and mild injection-site reactions	No specific rare event consistently identified in the reviewed sources	Not reported
*Givosiran*	siRNA	2019	AHP	ALAS1	GalNAc conjugated	SC	Injection-site reactions, nausea, fatigue, headache, and abdominal pain, laboratory abnormalities regarding liver and renal function	Anaphylaxis, hypersensitivity, pancreatitis	Liver, kidney
*Comirnaty*	mRNA	2021	COVID-19	SARS-CoV-2 spike protein	LNP	IM	Injection-site reactions, fatigue, headache, myalgia, chills, and fever	Myocarditis, pericarditis, anaphylaxis, ITP, Bell’s palsy, acute transverse myelitis, GBS, urticaria, morbilliform eruptions, aseptic meningitis	Cardiac, liver, brain
*Spikevax*	mRNA	2022	COVID-19	SARS-CoV-2 spike protein	LNP	IM	Injection-site reactions, fatigue, headache, myalgia, and fever	Compared to comirnaty: Lower rate of anaphylaxis, higher of non-anaphylactic hypersensitivity, cutaneous reactions, myocarditis and pericarditis	No specific major organ toxicity established in the reviewed sources
*mNEXSPIKE*	mRNA	2025	COVID-19	Prefusion-stabilized SARS-CoV-2 spike protein	LNP	IM	Comparable safety profile to spikevax	Comparable safety profile to spikevax	No specific major organ toxicity established in the reviewed sources
*mRESVIA*	mRNA	2024	RSV	RSV F-glycoprotein	LNP	IM	Injection-site pain, fatigue, headache, myalgia, and arthralgia	No cases of myocarditis or serious hypersensitivity reactions, GBS	No specific major organ toxicity established in the reviewed sources
*Nusinersen*	ASO	2016	SMA	SMN2 (splicing modulation)	Chemically modified ASO; no carrier-mediated delivery	IT	Lower back pain, gastrointestinal disturbances, headache	Fever, hypothermia, alopecia (reported less frequently)	No significant nephrotoxicity reported
*Eteplirsen*	ASO	2016	DMD	Dystrophin (exon 51 skipping)	PMO	IV	Procedural pain and headache, arthralgia, back pain, pain in the extremities, oropharyngeal pain, sprains, cough, nasal congestion, proteinuria, vomiting	Anaphylaxis	Rare nephrotoxicity
*Golodirsen*	ASO	2019	DMD	Dystrophin (exon 53 skipping)	PMO	IV	Headache, pyrexia, falls, abdominal pain, nasopharyngitis, cough, nausea, and vomiting	Hypersensitivity, anaphylaxis, infections or systemic septicemia	Rare nephrotoxicity
*Viltolarsen*	ASO	2020	DMD	Dystrophin (exon 53 skipping)	PMO	IV	Nasopharyngitis, cough, nasal congestion, flu-like symptoms, fever, headache, vomiting, diarrhea, rash, bruising	Limb edema, swelling of axillary lymph nodes	No significant renal or systemic toxicity reported
*Casimersen*	ASO	2021	DMD	Dystrophin (exon 45 skipping)	PMO	IV	Flushing, rash, contact dermatitis	Bacteremia, septic embolism, deep vein thrombosis	Low systemic toxicity
*Tofersen*	ASO	2023	ALS	SOD1	PMO	IT	Pain, fatigue, arthralgia, increased CSF white blood cells, neuralgia, myalgia, musculoskeletal stiffness	Myelitis, radiculitis, meningitis, papilledema	No significant hepatotoxicity and nephrotoxicity
*Inotersen*	ASO	2018	hATTR	TTR	Chemically modified ASO	SC	Diarrhea, nausea, urinary tract infections, falls, fatigue, vomiting, chills, injection site reactions, constipation, headache, syncope, dyspnea	Thrombocytopenia, hypertransaminasemia, hematologic and renal complications, ocular problems	Nephrotoxicity
*Eplontersen*	ASO	2023	ATTRv PV	TTR	GalNAc conjugated	SC	Reduction of vitamin A, vomiting, proteinuria, injection-site reactions, blurred vision, cataract	Atrioventricular heart block	No specific major organ toxicity established in reviewed sources

Abbreviations: AHP, acute hepatic porphyria; ALAS1, 5-aminolevulinate synthase 1; ALS, amyotrophic lateral sclerosis; ASO, antisense oligonucleotide; DMD, Duchenne muscular dystrophy; GalNAc, N-acetylgalactosamine; GBS, Guillain-Barré syndrome; hATTR, hereditary transthyretin amyloidosis; IM, intramuscular; IT, intrathecal; ITP, immune thrombocytopenia; IV, intravenous; LNP, lipid nanoparticle; mRNA, messenger RNA; PMO, phosphorodiamidate morpholino oligomer; RSV, respiratory syncytial virus; SC, subcutaneous; siRNA, small interfering RNA; SMA, spinal muscular atrophy; TTR, transthyretin; URI, upper respiratory infection.

A major milestone in the development of RNA therapeutics field was the work of Katalin Karikó and Drew Weissman, who were awarded the 2023 Nobel Prize in Physiology or Medicine for studying nucleoside base modifications and enabled the efficient design of mRNA vaccines against COVID-19 (The Nobel Prize in Physiology or Medicine 2023, n.d.). Their study, in 2005, demonstrated that the incorporation of modified nucleosides into mRNA molecules could prevent excessive activation of the immune system, thereby overcoming a critical obstacle in the application of RNA-associated therapies (Karikó et al., 2005). Subsequent work in 2008 further showed that the incorporation of pseudouridine enhances mRNA stability and translational efficiency, while reducing mRNA immunogenicity (Karikó et al., 2008). These landmark studies laid the foundation not only for the successful mRNA vaccine technology, but also expanded the development of RNA therapeutics.

Despite these advances, RNA therapeutics exhibit several limitations. As with all pharmacological interventions, therapeutic efficacy and safety profiles may vary considerably among platforms and patient populations. Several challenges remain, including inefficient extrahepatic delivery, which can elevate toxicity risks, immune activation induced by RNA molecules or lipid nanoparticle carriers, off-target gene silencing and organ-specific inflammatory toxicities (Pozdniakova et al., 2025). In addition, the rapid development and the relatively recent clinical introduction of currently approved RNA-related drugs resulted in limited long-term safety data and insufficient documentation of rare adverse events, highlighting the need for a collective and comprehensive evaluation across the RNA-based platforms (Saw and Song, 2024).

Most previous safety-focused reviews have examined individual RNA modalities, specific drug classes, a specific disease or particular delivery technologies. Accordingly, direct qualitative comparison of safety determinants across approved ASO, siRNA, and mRNA products remains limited. The present review addresses this gap by retaining drug-level clinical context while comparing how modality, molecular design, delivery strategy, administration route, and biodistribution may contribute to shared and distinct safety patterns.

## 2 Methods

This review was conducted to summarize and compare the adverse effects associated with currently FDA-approved RNA therapeutics, including siRNAs, mRNA-based therapeutics and ASOs. We performed a literature search using PubMed, Scopus, Web of Science, and Google Scholar databases for studies published up to June 2026. Search terms included combinations of “RNA therapeutics”, “RNA-based drugs”, “siRNA”, “antisense oligonucleotide”, “mRNA therapeutics”, “adverse effects”, “toxicity”, “safety” and the names of individual FDA-approved agents.

Eligible sources included clinical trials, extension studies, observational studies, pharmacovigilance analyses, systematic reviews, regulatory documents from the U.S. Food and Drug Administration (FDA) and European Medicines Agency (EMA) official websites, prescribing information, and relevant post-marketing safety reports. Publications not available in English, conference abstracts without sufficient safety data, duplicate reports, and preclinical studies without direct clinical relevance were excluded. For each therapeutic agent, information regarding approved indications, mechanism of action, route of administration, reported adverse effects, serious adverse reactions, and potential toxicity mechanisms was extracted. The collected data were organized according to therapeutic platform (siRNA, mRNA, and ASO therapeutics) and subsequently compared to identify common and distinct safety characteristics across RNA-based treatment modalities.

For the purposes of this review, only FDA-approved agents belonging to the three prespecified modalities (siRNA, mRNA, and ASO) and falling within the review scope at the June 2026 cut-off were included. The included products, along with their approval year, indication, target, delivery system, route of administration, and safety characteristics, are summarized in Table 1. Regulatory and prescribing-information sources were used to establish approved indications and labeled safety information, while clinical trials and extension studies were used to characterize adverse-event patterns in defined study populations. Pharmacovigilance and post-marketing reports were interpreted primarily as sources of rare or emerging safety signals rather than as proof of causality.

Because the included evidence sources differed in design, population, follow-up, and adverse-event reporting, no quantitative cross-study pooling or formal risk-of-bias grading was undertaken. When observations differed across evidence types, the terminology and causal interpretation of the original source were retained, and the strength and context of the evidence were considered qualitatively. No independent causality assessment of individual adverse events was performed. Events were described as adverse reactions only when the original clinical or regulatory source supported treatment attribution. Events with uncertain attribution were described as reported or observed adverse events without implying a direct causal relationship with the RNA therapeutic.

Frequency descriptors such as “common” and “rare” were retained only when they were explicitly reported or defined by the source used for the corresponding product; otherwise, numerical frequencies or neutral descriptive wording were preferred. “Serious” refers to events classified as serious in the underlying clinical or regulatory source and should not be interpreted as synonymous with high frequency or proven drug causality.

## 3 siRNA therapeutics

siRNA therapeutics act through the endogenous cellular mechanism of RNA interference (RNAi), which mediates post-transcriptional gene silencing. Once siRNA molecules enter the cell, they bind to the RISC (RNA-induced silencing complex) and the guide strand directs the specific sequence-binding and degradation of the complementary mRNA target (Figure 1A) (Alshaer et al., 2021; Wittrup and Lieberman, 2015). Although RNAi provides high specificity, off-target gene silencing, innate immune activation and delivery-associated toxicities, particularly in relation to carrier systems and organ-specific accumulation, cannot be excluded (Kanasty et al., 2013; Ebenezer et al., 2025). Safety interpretation therefore requires consideration of sequence-dependent RNAi effects together with delivery strategy (e.g., GalNAc conjugation *versus* LNP delivery), hepatic targeting, and administration route. The FDA-approved drugs based on the RNAi mechanism and their reported adverse effects are described below.

**FIGURE 1:**
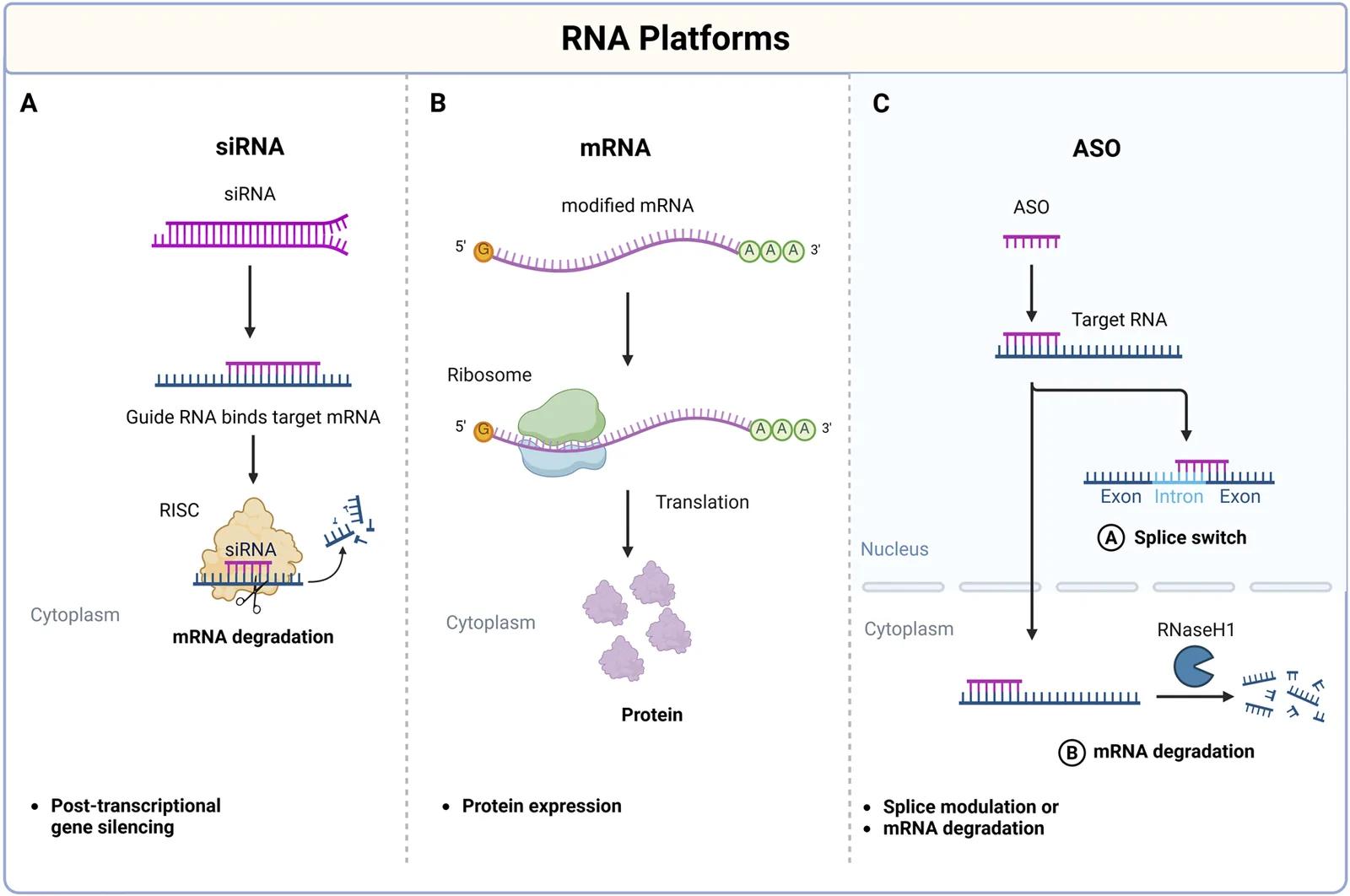
Schematic representation of the mechanisms of action of RNA-based therapeutic platforms. **(A)** siRNA therapeutics are incorporated into the RNA-induced silencing complex (RISC), where the guide strand directs sequence-dependent recognition and degradation of complementary mRNA. **(B)** mRNA therapeutics deliver modified mRNA for transient cytoplasmic translation; nucleoside modification reduces innate immune sensing and improves stability. **(C)** ASO therapeutics act through sequence-specific binding to RNA and can modulate pre-mRNA splicing or, depending on ASO design, promote RNase-H-dependent degradation of target RNA. The contribution of these mechanisms to safety is further modified by RNA chemistry, delivery system, administration route, and biodistribution.

### 3.1 Inclisiran

Inclisiran received FDA approval in December 2021 and has been developed for the management of hypercholesterolaemia, including both primary hypercholesterolaemia and mixed dyslipidaemia (Zhang et al., 2023). This encompasses patients with heterozygous familial hypercholesterolaemia (HeFH), a genetic disorder characterized by markedly elevated LDL-C (Low Density Lipoprotein Cholesterol) levels from an early age, as well as individuals with non-familial forms of the disease (Marrs and Anderson, 2024). In addition, inclisiran is widely used in patients with established atherosclerotic cardiovascular disease (ASCVD), including conditions such as coronary artery disease, previous myocardial infarction, ischemic stroke, and peripheral arterial disease (Huang et al., 2025). Inclisiran is an N-acetylgalactosamine-conjugated (GalNAc) siRNA that selectively targets PCSK9 mRNA (Figure 1A). Following subcutaneous administration, the GalNAc moiety mediates the uptake by hepatocytes through hepatic asialoglycoprotein receptors (Ray et al., 2023).

Inclisiran is generally well tolerated across multiple clinical trials and real-world studies (Zhang et al., 2023; Marrs and Anderson, 2024; Huang et al., 2025). Overall, the drug is well tolerated, with most adverse events being mild to moderate in severity and rarely leading to discontinuation. The most frequently reported adverse effects are injection-site reactions, including erythema, pain, rash, and swelling (Sabatine, 2019; Raal et al., 2024; Wilkinson et al., 2024). These reactions are generally mild, transient, and self-limiting and occur in approximately 5%–8% of patients receiving inclisiran (Wright et al., 2021). Despite being the most common treatment-related adverse event, they rarely require medical intervention. Other commonly reported adverse events include nasopharyngitis, upper respiratory tract infections, arthralgia, and headache (Wright et al., 2021; Li et al., 2026). However, these events occur at similar frequencies in both inclisiran and placebo groups, which limits confidence in attributing these events specifically to the drug (Basit et al., 2025). Their incidence is generally low and does not significantly affect treatment adherence. Regarding less common or rare adverse effects, hypersensitivity reactions have been reported, including mild allergic manifestations, such as rash. Severe reactions are considered rare, and no consistent reports of anaphylaxis have been identified (Zhang et al., 2023). In the same line, inclisiran has not been associated with significant hepatotoxicity, renal toxicity or muscle-related adverse effects, which are commonly observed with other lipid-lowering therapies, such as statins.

Overall, available evidence supports sustained tolerability with repeated administration, although continued long-term surveillance remains important as cumulative clinical exposure increases (Di Giacomo-Barbagallo et al., 2025; Lin et al., 2026).

### 3.2 Lumasiran

Lumasiran was approved by the FDA in 2020 for the treatment of primary hyperoxaluria type 1 (PH1), a rare and severe inherited metabolic disorder characterized by excessive hepatic oxalate production and progressive renal injury (Gang et al., 2023; Kang, 2024).

Following subcutaneous administration, lumasiran is delivered to the liver via conjugation with GalNAc and once internalized, it targets directly and silences the hepatic hydroxyacid oxidase 1 (HAO1) mRNA, which encodes the glycolate oxidase (Figure 1A). By inhibiting this enzyme, the drug reduces glyoxylate, and therefore oxalate production. This mechanism effectively lowers both urinary and plasma oxalate levels and addresses the cause of the disease rather than merely managing its clinical manifestations. Importantly, lumasiran is efficient across a broad patient population, including infants and young children, in whom early therapeutic intervention is critical to prevent irreversible organ damage (Gang et al., 2023). Moreover, lumasiran induces the sustained oxalate reduction with intermittent dosing. Lumasiran has generally demonstrated a favorable safety and tolerability profile in clinical trials and real-world use (Gang et al., 2023; Kang, 2024). Most adverse events are mild to moderate and rarely lead to treatment discontinuation. The most commonly reported adverse effects are injection-site reactions, which occur in approximately 30%–40% of patients and include pain, erythema, swelling, and pruritus at the site of administration (Gang et al., 2023; Hayes et al., 2023; Kang, 2024; Sellier-Leclerc et al., 2025). Other commonly observed adverse effects include abdominal pain, upper respiratory tract infections, and headaches. However, these are not always directly attributed to the drug, since they are typically reported in a minority of patients and their frequency is comparable to that observed in placebo groups, suggesting a limited causal relationship. Additionally, there is currently no strong evidence that link lumasiran to the development of significant hepatotoxicity, nephrotoxicity, or long-term systemic toxicity, which is particularly important given its hepatic mechanism of action.

Finally, long-term safety data remains somewhat limited, especially in very young patients and in those with advanced renal impairment. However, ongoing studies and post-marketing surveillance continue to support the overall safety of lumasiran across different age groups (Gang et al., 2023).

### 3.3 Nedosiran

The newer siRNA agent, nedosiran, approved by the FDA in 2023 for adults and children aged 9 years and older, differs from lumasiran in its mechanism of action. Rather than targeting glycolate oxidase, Nedosiran inhibits the hepatic lactate dehydrogenase A (LDHA) mRNA, thereby reducing oxalate production irrespective of the underlying genetic effect and offering potential efficacy across all PH subtypes (Figure 1A). Another difference to lumasiran is the delivery platform. While nedosiran uses GalXC technology, lumasiran utilizes the ESC-NGalNAc platform. In both cases, however, GalNAc targets the asialoglycoprotein receptor in hepatocytes facilitating hepatic drug uptake. Nedosiran is administered subcutaneously once per month and the most commonly reported adverse effects, occurring in more than 20% of patients, are injection-site reactions, as well as headache and nausea, while a single case of serious tachycardia has been also reported (Baum et al., 2023; Syed, 2023; Inc, 2025). However, an isolated event should not be interpreted as establishing a characteristic cardiovascular toxicity of nedosiran. Given its relatively recent approval, long-term safety data remains limited and continued post-marketing surveillance is important for characterizing uncommon and long-latency adverse events. Currently complete clinical trials have included pediatric participants of 2 years old, while ongoing clinical trials are further evaluating its safety in children from birth to 11 years of age (Sas et al., 2025).

### 3.4 Patisiran

Patisiran is the first FDA-approved siRNA agent, approved in 2018, and was developed for the treatment of hereditary transthyretin-mediated amyloidosis with polyneuropathy (hATTR), a progressive and often life-threatening condition characterized by the deposition of misfolded TTR in peripheral nerves and other organs in adults (Adams et al., 2018). It targets directly the TTR mRNA and is based on lipid nanoparticle (LNP) delivery system (Figure 1A).

Patisiran has generally demonstrated a favorable safety profile, however, several adverse events have been consistently reported. The most common adverse reactions include infusion-related reactions, occurring in approximately 19% of treated patients despite premedication (Adams et al., 2018; Zhang et al., 2021; Karimi et al., 2024). These reactions may present flushing, nausea, abdominal pain, headache, arthralgia, back pain, dyspnea, chest discomfort, and transient blood pressure changes. Upper respiratory tract infections, nasopharyngitis, influenza-like illness, peripheral edema, and fatigue have also been frequently observed (Urits et al., 2021; Karimi et al., 2024). Because TTR serves as a carrier protein for retinol-binding protein, prolonged TTR suppression may lead to reductions in serum vitamin A levels, necessitating vitamin A supplementation during treatment (Adams et al., 2018; Urits et al., 2021). Serious treatment-related adverse events are uncommon, and clinically significant hepatotoxicity, nephrotoxicity, or severe immunogenicity have not been consistently reported.

### 3.5 Vutrisiran

Vutrisiran was approved in June 2022 and represents a next-generation evolution of patisiran (Keam, 2022). Similar to patisiran, vutrisiran targets a conserved sequence of TTR mRNA, resulting in potent and sustained suppression of TTR production (Figure 1A) (Keam, 2022).

Unlike patisiran, vutrisiran uses GalNAc-mediated hepatocyte targeting and is administered subcutaneously every 3 months without an LNP carrier or intravenous infusion. This delivery strategy avoids the infusion-related adverse-event pattern associated with intravenous LNP administration and illustrates how delivery technology and route can modify the safety profile of therapeutics acting through the same RNAi mechanism (Zhang et al., 2021; Urits et al., 2021; Keam, 2022; Karimi et al., 2024). This comparison should not, however, be interpreted as evidence that GalNAc conjugation is intrinsically safer across all RNA therapeutics, because product-specific chemistry, exposure, dosing, and clinical context also differ. The most commonly reported adverse effects of vutrisiran include arthralgia, pain in extremities, dyspnea, falls, peripheral edema, and mild injection-site reactions (Zhang et al., 2021; Karimi et al., 2024). Similar to patisiran, reductions in circulating vitamin A levels have been observed and supplementation is recommended during treatment (Brown et al., 2026). Overall, adverse events are predominantly mild to moderate in severity and rarely result in treatment discontinuation.

### 3.6 Givosiran

Givosiran was approved by the FDA in 2019 for the treatment of acute hepatic porphyria (AHP) (Dickey and Leaf, 2024). It is administered subcutaneously as a GalNAc-conjugated siRNA and targets aminolevulinate synthase 1 (ALAS1), the rate-limiting enzyme in hepatic heme synthesis, thereby reducing the accumulation of the neurotoxic intermediates aminolevulinic acid and porphobilinogen (Figure 1A) (Majeed et al., 2022). Clinical efficacy was demonstrated in the phase III ENVISION trial, which showed a significant reduction in the annualized rate of porphyria attacks compared to placebo (Majeed et al., 2022; Sardh and Harper, 2022). Givosiran is generally well tolerated, with most adverse events being mild to moderate in severity. The most commonly reported adverse effects include injection-site reactions, nausea, fatigue, headache, and abdominal pain (Majeed et al., 2022; Dickey and Leaf, 2024). However, clinically relevant laboratory abnormalities have also been reported, including elevations in liver enzymes, renal function abnormalities, and hyperhomocysteinemia (Majeed et al., 2022; Dickey and Leaf, 2024; Xu et al., 2026). Their interpretation requires consideration of the intended hepatic pharmacological effect, alterations in heme-pathway metabolism, underlying acute hepatic porphyria, concomitant treatments, and duration of exposure.

## 4 mRNA therapeutics

Messenger RNA (mRNA) therapeutics use synthetic mRNA to direct host cells to transiently produce target proteins without genomic integration (Figure 1B) (Ige et al., 2025). Their clinical translation was enabled by nucleoside modification strategies, most notably the substitution of uridine with pseudouridine, which reduces mRNA immunogenicity and improves stability, as well as lipid nanoparticle (LNP) delivery systems that protect mRNA from degradation and facilitate intracellular uptake (Karikó et al., 2008; Pardi et al., 2018). To date, four mRNA-based therapeutics have received full FDA approval, three COVID-19 vaccines, named comirnaty, spikevax, and mNEXSPIKE and one RSV vaccine, named mRESVIA. For mRNA products, the safety profile reflects both the encoded antigen/protein and the formulation, with LNP components, innate immune sensing, dose, and intramuscular administration contributing differently to local, systemic, and rare immune-mediated events.

### 4.1 Comirnaty

Comirnaty (Tozinameran, BNT162b2) is an mRNA vaccine encoding the SARS-CoV-2 spike antigen, administered intramuscularly for the prevention of COVID-19 (Figure 1B). It received full FDA approval in August 2021 for individuals aged 65 years and older, or high risk 5 years and older and is subsequently updated annually for circulating variants. The most reported adverse effects are local and systemic reactions consistent with immune activation: injection-site pain, fatigue, headache, myalgia, chills, and fever, typically resolving within one to two days and more pronounced after the second dose. Rare but clinically significant adverse events include myocarditis and pericarditis, occurring predominantly in adolescent and young adult males within the first week after the second dose (approximately 12.6 cases/10^6^ s doses) (Matar et al., 2022; Hromić-Jahjefendić et al., 2023). Anaphylaxis has also been reported. Hypersensitivity to LNP components, including PEG-containing lipids, has been investigated as a potential mechanism, although the immunological mechanisms underlying individual reactions may be heterogeneous (Jaggers and Wolfson, 2023). Immune thrombocytopenia and neurological events, including Bell’s palsy, transverse myelitis, Guillain–Barré syndrome, and aseptic meningitis, have been described in pharmacovigilance reports and case-based literature; however, reporting after vaccination does not by itself establish causality, and the strength of evidence differs among individual events (Gan et al., 2022; Oueijan et al., 2022; Kato et al., 2023; Atefi et al., 2024). Although sporadic severe cases have been reported, large pharmacovigilance analyses indicate very low reporting rates, and a definitive causal relationship has not been fully established. Clinical presentation typically includes severe headache, photophobia, and CSF (Cerebrospinal Fluid) pleocytosis without identifiable pathogens. A consistent female predominance has been observed for neurological and hypersensitivity adverse events, considered multifactorial in etiology (García-Grimshaw et al., 2021; Koh et al., 2021).

### 4.2 Spikevax

Spikevax (mRNA-1273) encodes the same SARS-CoV-2 spike antigen and is delivered via intramuscular LNP injection (Figure 1B). It received full FDA approval in January 2022 and is currently approved for individuals aged 6 months and older who are at high risk for severe COVID-19. Its adverse effect profile is broadly similar to comirnaty, with injection-site reactions, fatigue, headache, myalgia, and fever being the most common events (Oueijan et al., 2022). Key differences from comirnaty include a lower rate of anaphylaxis (approximately 3.42/10^6^ doses vs. 9.31 for comirnaty), but a higher rate of non-anaphylactic hypersensitivity and cutaneous reactions, including a higher incidence of delayed large local reactions (DLLRs), which occurred in 94% of DLLR cases across both vaccines (Alhumaid et al., 2021). Myocarditis and pericarditis are rare but clinically important safety considerations for mRNA COVID-19 vaccines, occurring most prominently in adolescent and young adult males shortly after vaccination. Some observational studies have reported higher myocarditis rates after mRNA-1273 than after BNT162b2; however, comparisons should account for differences in age distribution, dose, dosing interval, vaccine schedules, surveillance methods, and population characteristics and should not be interpreted as demonstrating an intrinsic platform-level difference (Hromić-Jahjefendić et al., 2023). Anaphylactic, non-anaphylactic hypersensitivity, and cutaneous reactions have also been reported (Alhumaid et al., 2021). Pharmacovigilance and trial reports of other rare events, including facial paralysis, warrant surveillance but do not necessarily establish a causal association with vaccination (El Sahly et al., 2021; Urdaneta et al., 2024). Facial paralysis, including Bell’s palsy, was reported in eight vaccine recipients *versus* three placebo recipients in the pivotal phase 3 trial, and is listed in the official prescribing information, though current evidence is insufficient to establish a causal relationship (El Sahly et al., 2021). Facial swelling in individuals with a prior history of dermal filler injections has also been recognized as a rare adverse event (fewer than 1 in 1,000) by the European Medicines Agency, reflecting a localized immune-mediated hypersensitivity response (Cirillo, 2021).

### 4.3 mNEXSPIKE

mNEXSPIKE (mRNA-1283) is a next-generation COVID-19 mRNA vaccine and was approved by FDA in May 2025, indicated for adults aged 65 and older, and individuals aged 12–64 with at least one high-risk underlying condition (Chalkias et al., 2025). It uses a shorter mRNA construct encoding a modified prefusion-stabilized spike protein and is administered at a lower dose than spikevax (Figure 1B).

Clinical trial data indicate a comparable safety profile to spikevax, with injection-site reactions, fatigue, and headache as the most frequently reported adverse events. As with the other approved COVID-19 mRNA vaccines, myocarditis and pericarditis remain important safety considerations predominantly in adolescent and young adult males aged 12–24 years. Long-term safety data remain limited given its recent approval, and ongoing pharmacovigilance is required (Chalkias et al., 2025; Domachowske et al., 2026).

### 4.4 mRESVIA

mRESVIA (mRNA-1345) is the first approved non-COVID-19 mRNA therapeutic, indicated for the prevention of lower respiratory tract disease caused by Respiratory Syncytial Virus (RSV) (Ma et al., 2025; Mayer et al., 2026). It received initial FDA approval in May 2024 for adults aged 60 and older, with an expanded indication for at-risk adults aged 18–59 approved in June 2025. It encodes the RSV F-glycoprotein and is delivered as a single intramuscular dose (Figure 1B).

Adverse effects reported in clinical trials are predominantly mild to moderate and include injection-site pain, fatigue, headache, myalgia, and arthralgia. No notable excess of myocarditis or serious hypersensitivity was identified in the pivotal clinical evidence summarized here; however, the smaller cumulative exposure and comparatively limited post-marketing follow-up mean that uncommon events cannot yet be characterized with the same precision as for the more widely administered COVID-19 mRNA vaccines (Ma et al., 2025; Mayer et al., 2026). Continued surveillance is therefore required as exposure increases. As with other LNP-based mRNA vaccines, hypersensitivity reactions related to PEGylated lipid components remain a theoretical concern (Ma et al., 2025; Mayer et al., 2026). Long-term safety surveillance is ongoing and essential, given the relatively recent approval and the targeted age group.

## 5 ASO therapeutics

Antisense Oligonucleotide (ASO) therapeutics are emerging as a promising new class of treatment approaches, especially for diseases that have been challenging to tackle with conventional ways. ASOs are single-stranded synthetic deoxyribonucleotide sequences (typically 16–21 nucleotides) that can be designed to switch off or adjust the production of specific disease-related proteins by base-pairing to the targeted cellular mRNA to initiate their action and leverage their therapeutic potential at the mRNA level (Figure 1C) (Liang and Zhang, 2026). The latest innovations in chemical alterations, in combination with breakthroughs in drug delivery, have led to the development and approval of 16 antisense oligonucleotides with strong pharmaceutical potential, however, not all of them are currently in use. To date, 8 FDA-approved ASOs are in clinical use, including Nusinersen, Eteplirsen, Inotersen, Golodirsen, Viltolarsen, Casimersen, Tofersen, and finally Eplontersen (Chiriboga et al., 2016; Moriyama et al., 2026). ASO safety is particularly heterogeneous because splice-modulating and RNase-H-active designs, backbone and sugar chemistry, tissue distribution, and intrathecal, intravenous, or subcutaneous administration can produce distinct toxicity and procedure-related patterns.

### 5.1 Nusinersen

Nusinersen was approved by the FDA in December 2016. This drug is the first effective gene-modifying treatment for all types of Spinal Muscular Atrophy (SMA) (Qiu et al., 2022). It functions through splicing modulation of SMN2 pre-mRNA rather than mRNA degradation or protein replacement (Figure 1C) (Chiriboga et al., 2016). Unlike mRNA therapeutics and some siRNAs that require nanoparticle-based delivery systems, nusinersen is administered as a chemically modified ASO without carrier-mediated delivery via intrathecal injection directly into CSF due to poor blood-brain barrier penetration. Most studies conducted on this drug involve infants and children, as the most severe forms of SMA (Types 1–3) typically begin at these ages. It is important to note that most studies involve a pediatric population, and it appears that, overall, more boys/men than girls/women participate in most studies.

In the pediatric population, the most common side effect is lower back pain following a lumbar puncture (Li et al., 2024). This is followed by gastrointestinal disturbances, which were of short duration and manifested primarily as vomiting. Respiratory tract infections and pulmonary infections occur with similar frequency; these follow the puncture and are often attributed in part to the underlying disease or the patients’ general condition (Acsadi et al., 2021; Finkel et al., 2026). Less commonly, fever, hypothermia, or even a skin rash may be observed in some cases. However, these symptoms appear to be resolved spontaneously within a few hours. More rarely, alopecia may occur, ranging in severity from mild to more severe, which subsides partially or completely after the patients’ general condition improves. Furthermore, death may occur, although in most cases this has been attributed to conditions associated with the progression of the underlying disease (He et al., 2026).

In adults, the most common side effect was also lower back pain resulting from the intrathecal administration of nusinersen. Headaches occurred fairly frequently in patients and appeared to be more common in women than men (Walter et al., 2019; Zaidman et al., 2026). Additionally, some patients experience nausea (Claborn et al., 2019; Zaidman et al., 2026). A substantial proportion of commonly reported events, particularly headache and low-back pain, are compatible with the intrathecal administration procedure. Furthermore, certain events, such as infections and respiratory complications, may be related to the underlying disease, making it difficult to clearly distinguish between adverse drug reactions and manifestations of the disease (Claborn et al., 2019). Serious adverse drug reactions appear to be rare, and no significant issues of nephrotoxicity or other serious laboratory abnormalities have emerged (He et al., 2026). Furthermore, the safety profile remains consistent across different age groups, supporting the drug’s good tolerability in a wide range of patients.

### 5.2 Eteplirsen

Eteplirsen received approval by the FDA in September 2016 for the treatment of patients with Duchenne muscular dystrophy (DMD) amenable to exon 51 skipping of the dystrophin pre-mRNA (Figure 1C) (Moriyama et al., 2026). DMD is a sex-linked, recessive neuromuscular disorder that affects male infants and is characterized by progressive muscle degeneration. Eteplirsen is delivered systemically as a phosphorodiamidate morpholino oligomer (PMO) without a nanoparticle carrier (Mendell et al., 2013).

Very common adverse reactions associated with Eteplirsen include procedural pain and headache, suggesting a significant contribution from the administration procedure (Zhang et al., 2026). Common adverse events include arthralgia, back pain, pain in the extremities, oropharyngeal pain, sprains, nasopharyngitis, upper respiratory tract infections, cough, nasal congestion, proteinuria, and vomiting (Alfano et al., 2019; Sheikh and Yokota, 2022; Mercuri et al., 2023; Iff et al., 2024). Moderately frequent events include balance disorders, upper abdominal pain, prolonged activated partial thromboplastin time, elevated C-reactive protein, dyspepsia, hypokalemia, muscle spasms, and administration-related reactions, such as pain or bleeding at the catheter site (Alfano et al., 2019; Sheikh and Yokota, 2022). Finally, fever is observed less frequently.

Interpretation of these events requires consideration of both treatment exposure and the clinical context of DMD. Procedural pain, catheter-site symptoms, and some administration-related reactions may plausibly reflect repeated intravenous access rather than sequence-specific PMO toxicity (Sheikh and Yokota, 2022; Konieczny, 2024). First, a significant proportion of adverse events, such as procedural pain and headache, are related to the intravenous infusion procedure and the use of a venous catheter, leading to local irritation and an inflammatory reaction. Likewise, musculoskeletal symptoms and respiratory infections occur commonly in patients with DMD and cannot automatically be attributed to eteplirsen. Hypersensitivity reactions have nevertheless been reported, and renal surveillance remains relevant for PMO therapeutics because of renal handling of oligonucleotides and safety findings observed with some members of this class (Alfano et al., 2019; Sheikh and Yokota, 2022; Mercuri et al., 2023; Iff et al., 2024).

### 5.3 Golodirsen

Golodirsen received FDA approval in December 2019 for the treatment of DMD in patients with mutations amenable to exon 53 skipping of the dystrophin pre-mRNA (Figure 1C) (Moriyama et al., 2026). Golodirsen is a PMO ASO administered by weekly intravenous infusion. It promotes exon 53 skipping during pre-mRNA splicing, thereby restoring the reading frame of the dystrophin transcript and enabling the production of a truncated but functional dystrophin protein.

Golodirsen has generally demonstrated an acceptable safety profile, with most adverse events being mild to moderate in severity (Alhamadani et al., 2022). The most frequently reported adverse effects include headache, pyrexia, falls, abdominal pain, nasopharyngitis, cough, nausea, and vomiting. Additional adverse events reported less frequently include injection-site pain, diarrhea, dizziness, rhinitis, pharyngitis, influenza-like illness, seasonal allergies, bruising, sprains, skin abrasions, constipation, tachycardia, ear infections, and bone fractures. Hypersensitivity reactions have also been observed, including rash, pruritus, dermatitis, urticaria, and skin exfoliation (Sabrina Haque et al., 2024). An important safety concern about golodirsen is the potential risk of renal toxicity. Although clinically significant nephrotoxicity has not been established consistently in treated patients, renal tubular abnormalities were identified in preclinical studies, supporting recommendations for renal-function monitoring during treatment (Alhamadani et al., 2022; Migliorati et al., 2022). Overall, available clinical evidence suggests that golodirsen is generally well tolerated, although continued long-term monitoring remains important, particularly regarding renal safety.

### 5.4 Viltolarsen

Viltolarsen was approved by the FDA in 2020 for DMD caused by exon 53 skipping of dystrophin pre-mRNA (Figure 1C) and is delivered as a PMO intravenously (Moriyama et al., 2026). In the majority of the studies, the participants were children, while some others included adolescent boys up to about 19 years of age (Clemens et al., 2020; 2022; 2023; Komaki et al., 2020; Sabrina Haque et al., 2024; Harper et al., 2024; Funato et al., 2025). Nearly all participants experienced at least one treatment-emergent adverse event; however, none of these events necessitated discontinuation of treatment, dose reduction, or permanent withdrawal of the drug. The most reported adverse events included nasopharyngitis, upper respiratory tract infections, cough, nasal congestion, flu-like symptoms, fever, headache, vomiting, diarrhea, rash, and bruising. Falls and musculoskeletal injuries were also frequently reported, which are likely related to the underlying muscle weakness of DMD rather than direct pharmacological toxicity. Administration-related events such as extravasation and local edema are plausibly related to repeated intravenous access, whereas falls and musculoskeletal injuries must be interpreted in the context of the underlying DMD phenotype. Clinically significant nephrotoxicity has not been consistently demonstrated in the available studies; nevertheless, renal monitoring remains relevant because preclinical renal findings have been reported for members of the PMO class (Clemens et al., 2020; 2022; 2023; Komaki et al., 2020; Funato et al., 2025).

### 5.5 Casimersen

Casimersen was approved by the FDA in 2021 to treat DMD patients who are prone to exon 45 skipping of dystrophin pre-mRNA (Figure 1C) (Moriyama et al., 2026). Reported adverse events with casimersen include rash, contact dermatitis, flushing, headache, vomiting, and administration-related symptoms, while hypersensitivity reactions have also been described (Wagner et al., 2021). Vascular and infectious complications, including bacteremia, septic embolism, and deep-vein thrombosis, have been reported in the clinical setting. Repeated intravenous access, underlying disease, comorbidities, and other clinical factors may contribute to such events. Available clinical data have not demonstrated a consistent pattern of major renal, cardiac, or hematological toxicity, although continued surveillance remains warranted given the limited size and duration of the available clinical experience (Sabrina Haque et al., 2024).

### 5.6 Tofersen

Tofersen received FDA approval in 2023 for the treatment of amyotrophic lateral sclerosis (ALS) by targeting and degrading human SOD1 mRNA (Figure 1C) (van Roon-Mom et al., 2023; McGuigan and Blair, 2025). It is delivered via intrathecal injection into the CSF/spinal canal and among the main related adverse effects reported are pain, fatigue, arthralgia, increased CSF white blood cell count, neuralgia, myalgia and musculoskeletal stiffness. Treatment discontinuation occurred in 20% of the participants and 15% of the patients experienced lethal adverse effects; however they were all related to already existing comorbidities or disease deterioration (Miller et al., 2022; Lovett et al., 2025). Among the most serious adverse effects which have been reported are myelitis, radiculitis, aseptic meningitis, and papilledema (Lovett et al., 2025). Their interpretation requires particular caution because tofersen is delivered directly into the cerebrospinal fluid and repeated lumbar puncture, local CNS exposure, inflammatory responses, and progression of SOD1-ALS may all contribute to the observed clinical picture. Oligonucleotides can interact with innate immune and complement pathways depending on their sequence and chemistry, providing biological plausibility for inflammatory effects; however, this does not establish off-target hybridization as the cause of individual neurological events (Everett and Bucelli, 2024; Xiao et al., 2026). According to the EMA and FDA reports, no clinical safety or efficacy has been established to the paediatric population and sex-related differences in drug pharmacodynamics have not been revealed with clear evidence or trends in drug safety. Continued clinical and pharmacovigilance monitoring is therefore required to better characterize the mechanisms and incidence of these uncommon, serious events.

### 5.7 Inotersen

Inotersen was approved both in Europe and USA in 2018 and it is used to treat polyneuropathy in adults with stage 1 or 2 hereditary transthyretin amyloidosis (hATTR-PN) via targeting TTR mRNA (Figure 1C). It is delivered as a chemically modified ASO alone subcutaneously and once per week (Benson et al., 2018; Dasgupta et al., 2020).

In most clinical studies, the participants were adults, while in a smaller number of studies the population consisted of older adults. However, in both age groups, men outnumbered women. The most common side effects, which occur in a significant proportion of the population, are mainly gastrointestinal, systemic, and infectious. Specifically, they include diarrhea, nausea, urinary tract infections, falls, fatigue, and vomiting. Sometimes patients experience chills, peripheral edema, pain and erythema at the injection site, constipation, headache, as well as syncope and dyspnea. Most of these are mild to moderate and often self-limiting. Less frequently, but with clinical significance, thrombocytopenia and glomerulonephritis occur. Thrombocytopenia is the most significant complication and may lead to bleeding episodes. Adverse effects such as elevated liver transaminases and ocular problems occur rarely (Brannagan et al., 2022; Koch et al., 2026).

The safety profile of inotersen requires distinction between expected pharmacodynamic effects and clinically important toxicities. Reduction in circulating TTR can alter retinol transport and therefore vitamin A homeostasis, reflecting the intended pharmacological action of TTR suppression. In contrast, severe thrombocytopenia and glomerulonephritis represent important adverse reactions requiring dedicated laboratory monitoring. Immune-mediated mechanisms have been proposed for some of these complications, particularly thrombocytopenia, although the mechanisms underlying individual events may be multifactorial (Narayanan et al., 2020; Brannagan et al., 2022). Injection-site reactions and nonspecific systemic symptoms represent a separate safety category related to subcutaneous administration and systemic exposure. This distinction illustrates why ASO safety should not be considered uniform across the class (Narayanan et al., 2020; Dasgupta et al., 2020). In summary, the safety profile of inotersen is characterized by the occurrence of frequent but mostly mild to moderate adverse reactions. Although less common, hematologic and renal complications are potentially serious and require close laboratory monitoring during treatment.

### 5.8 Eplontersen

Eplontersen was approved in 2023 by the FDA for the treatment of ATTRv-PN and is now also studied in ATTR cardiomyopathy. It targets and reduces both the mutant and wild type TTR in the liver (Figure 1C). The lower levels of TTR reduce the dissociation and misfolding of the tetramers of TTR and their deposition in the nerves and cardiac tissue, which is the main cause behind the disease. It is a GalNAc-conjugated drug that is subcutaneously administered once per month (Conceição et al., 2024; Masri et al., 2024).

Among the most common adverse effects reported are vomiting, proteinuria, injection-site reactions, blurred vision and cataract, while reduction of serum vitamin A are expected as a pharmacodynamic consequence of lowering circulating TTR, which transports retinol-binding protein (Coelho et al., 2023; Wixner et al., 2025). Serious adverse reactions of atrioventricular (AV) heart block were also reported in 2% of treated patients, but their occurrence should be interpreted in the context of underlying ATTR amyloidosis. No statistically significant differences in sex-related adverse effects were reported after eplontersen administration and no guidelines have been established or published so far regarding paediatric data.

## 6 Cross-platform safety comparison

Cross-platform comparison of RNA therapeutics indicates that adverse-event profiles cannot be adequately explained by RNA modality alone. Although ASOs, siRNAs, and mRNAs differ fundamentally in molecular structure and mechanism of action, the clinical safety profile of an individual product emerges from the interaction between sequence and chemical composition, delivery technology, route and frequency of administration, biodistribution, target-tissue exposure, clearance, and the characteristics of the treated population. Platform identity therefore remains an important determinant of safety but should be considered within this broader pharmacological and clinical framework rather than as an independent predictor of toxicity (Juliano, 2016; Pardi et al., 2018; Roberts et al., 2020; Zhu et al., 2022).

Delivery strategy and route of administration provide particularly informative examples of this interaction. GalNAc conjugation promotes the uptake of oligonucleotide therapeutics by hepatocytes, thereby increasing hepatic selectivity and reducing exposure of non-target tissues. However, the resulting concentration of drug within the liver makes hepatic exposure and hepatobiliary handling relevant to safety interpretation. In contrast, LNPs protect RNA cargo from degradation and facilitate intracellular delivery, but the formulation itself may contribute to inflammatory responses, complement activation, or hypersensitivity independently of the biological activity of the RNA cargo (Kulkarni et al., 2019; Hou et al., 2021).

Administration route adds another layer of complexity. Subcutaneously administered GalNAc-conjugated agents are frequently associated with local injection-site reactions, intravenous products may produce infusion- or vascular-access-related events, and intrathecal ASOs expose the central nervous system directly while also introducing adverse events related to lumbar puncture. Thus, apparently platform-associated toxicities may in some cases reflect delivery or administration characteristics rather than the RNA modality itself. This distinction is illustrated by therapeutics directed against the same molecular target. Patisiran and vutrisiran both suppress hepatic TTR expression through RNA interference, but patisiran is delivered intravenously in an LNP formulation whereas vutrisiran is a subcutaneously administered GalNAc-conjugated siRNA. Their different safety patterns therefore demonstrate how formulation and route can modify the clinical expression of toxicity within the same therapeutic platform (Zhang et al., 2021; Keam, 2022; Karimi et al., 2024). Similarly, the PMO ASOs eteplirsen, golodirsen, viltolarsen, and casimersen share a related chemical architecture and intravenous route, yet differences in reported adverse events and monitoring requirements indicate that chemistry alone does not determine the safety profile. Product-specific exposure, disease context, treatment duration, and the strength of the available clinical evidence must also be considered (Alhamadani et al., 2022).

Innate immune activation represents a potential shared biological mechanism across RNA therapeutic classes, although its molecular basis and clinical consequences differ according to modality and formulation. Exogenous RNA and oligonucleotide structures may interact with pattern-recognition pathways, whereas delivery components such as PEG can independently contribute to inflammatory or hypersensitivity responses (Pardi et al., 2018; Hou et al., 2021; Zhu et al., 2022).

In the same line, biodistribution and clearance further determine which organs are exposed and therefore which toxicities require particular surveillance. The liver represents a major site of exposure for several RNA therapeutics because of both physiological clearance mechanisms and intentional hepatocyte targeting, particularly for GalNAc-conjugated siRNAs and ASOs. Renal handling is relevant for several oligonucleotide therapeutics and is influenced by molecular size, charge, protein binding, chemical modification, and tissue uptake. For the PMO therapeutics used in DMD, clinically significant nephrotoxicity has not been consistently demonstrated, but renal surveillance remains relevant in view of preclinical renal findings and oligonucleotide disposition. Neurological safety requires a different interpretative framework for intrathecal agents such as nusinersen and tofersen because direct exposure of the cerebrospinal fluid and neural tissues occurs together with procedural risks associated with repeated lumbar puncture. In these settings, neurological or systemic deterioration may also arise from the underlying progressive disease, emphasizing the importance of distinguishing pharmacologically plausible toxicity from procedure-related and disease-related events whenever the available evidence permits.

Repeated administration adds a temporal dimension to these safety determinants. Many approved RNA therapeutics require chronic or repeated dosing, and cumulative exposure may alter tissue concentrations, clearance, immune responses, or organ-specific effects in ways that are not fully captured by clinical trials of limited duration. The relevance of repeated exposure is expected to vary according to chemistry, formulation, dose, dosing interval, route, biodistribution, and tissue persistence rather than representing a uniform property of RNA therapeutics. Long-term extension studies and post-marketing surveillance are therefore particularly important for identifying delayed, cumulative, or rare hepatic, renal, hematological, immunological, or neurological events, especially for recently approved products and populations with limited clinical exposure.

Additionally, sequence-dependent off-target effects are mechanistically distinct among platforms. SiRNAs may suppress partially complementary transcripts through RISC-mediated interactions, whereas ASOs can hybridize with non-targeted RNA sequences or develop nonspecific interactions. For currently approved mRNA products, which in the present review consist of vaccines, safety considerations are more closely related to innate immune sensing, LNP formulation, expression of the encoded antigen, dose, and biodistribution than to classical RNAi-like off-target gene silencing.

Taken together, the available evidence supports a multifactorial and product-specific model of RNA therapeutic safety. Modality establishes the fundamental pharmacological mechanism, but chemistry, sequence, delivery system, route, biodistribution, target tissue, repeated exposure, and disease context collectively determine how toxicity is expressed clinically. Accordingly, the comparisons presented in Tables 1 and 2 should be interpreted as mechanistically informed qualitative comparisons rather than quantitative head-to-head estimates of comparative risk. This framework also argues against classifying one RNA platform as intrinsically safer or more toxic than another and instead supports safety assessment at the level of the individual therapeutic, its formulation, its route of administration, and its intended patient population.

**TABLE 2:** Cross-platform framework for interpreting adverse-event patterns in FDA-approved RNA therapeutics.

Safety determinant	siRNA	mRNA	ASO
Representative molecular action	RISC-mediated target-mRNA silencing	Transient translation of an encoded protein/antigen	Splice modulation or target-RNA degradation, depending on ASO design
Key chemistry considerations	Backbone/ribose chemistry and conjugation can influence stability, RISC activity, biodistribution and immune recognition	Modified nucleosides can reduce innate sensing and improve stability/translation	Backbone and sugar chemistry can influence stability, protein binding, immune recognition and tissue accumulation
Major delivery patterns	GalNAc-conjugated SC products or LNP-based IV delivery	LNP-based IM administration in currently approved products included here	Carrier-free or conjugated products; IT, IV or SC administration
Prominent administration-related patterns	Injection-site reactions with SC products; infusion-related reactions with IV LNP delivery	Local injection-site reactions and short-lived systemic reactogenicity	Lumbar-puncture complications for IT products; infusion/catheter reactions for IV products; injection-site reactions for SC products
Immune/inflammatory considerations	Innate immune activation and delivery-related immune effects; LNP-related reactions for patisiran	Innate sensing and LNP-associated inflammatory/hypersensitivity mechanisms; rare immune-mediated events require product-specific interpretation	Sequence/chemistry-dependent immune stimulation; hypersensitivity or complement-related effects may occur for some products
Organ-specific considerations	Hepatic/metabolic findings are especially relevant for hepatocyte-targeted agents; renal abnormalities reported for selected products	Cardiac and immune-mediated rare events are product/context specific; long-term organ toxicity remains under surveillance	Renal, hematological and neurological findings vary by chemistry, biodistribution, target and route
Off-target interpretation	RISC-mediated silencing of partially complementary transcripts	Classical RNAi-like off-target silencing is not the principal mechanism; encoded-product, innate-sensing and formulation effects are more relevant	Unintended hybridization or nonspecific chemistry-dependent interactions
Comparative interpretation	Product-specific; strongly modified by conjugation/delivery and hepatic targeting	Product-specific; strongly influenced by formulation, dose, immune context and target antigen/protein	Product-specific; cannot be attributed to platform alone

Abbreviations: IM, intramuscular; IT, intrathecal; IV, intravenous; SC, subcutaneous.

## 7 Conclusion

The clinical development of RNA therapeutics has established siRNAs, ASOs, and mRNAs as therapeutically important modalities across genetic, metabolic, cardiovascular, infectious, and neurodegenerative diseases (Roberts et al., 2020; Zhu et al., 2022). Analysis of the 18 FDA-approved products included in this review demonstrates that their safety profiles contain both shared and modality-specific features but also shows that platform classification alone provides an incomplete explanation for the adverse events observed clinically. Instead, safety reflects the interaction of molecular mechanism with sequence and chemical design, delivery technology, administration route, biodistribution, tissue exposure, clearance, treatment duration, and patient- and disease-specific factors. Therefore, safety monitoring should be tailored to the pharmacological and delivery characteristics of individual products, including relevant hepatic, renal, hematological, immunological, neurological, or administration-related risks. At the same time, adverse events must be interpreted in relation to therapeutic benefit. Monitoring requirements or manageable toxicities may have a different clinical significance in progressive or life-threatening genetic disease than rare serious events associated with preventive interventions administered to otherwise healthy populations. Risk–benefit assessment should therefore remain specific to the therapeutic, indication, target population, and available treatment alternatives rather than being extrapolated from the RNA platform.

Several limitations of the present evidence should, however, temper the interpretation of these cross-platform comparisons. This narrative review integrates evidence from clinical trials, extension and observational studies, regulatory documents, prescribing information, and pharmacovigilance analyses that differ substantially in population characteristics, sample size, adverse-event definitions, ascertainment methods, comparator groups, and duration of follow-up. Consequently, reported event frequencies cannot be assumed to be directly comparable across products. Pharmacovigilance data are particularly valuable for identifying rare or emerging safety signals but are susceptible to under-reporting, missing denominator information, duplicate reports, and confounding by indication and generally cannot establish causality. Publication and selective-reporting bias may further influence the available safety literature. Moreover, long-term exposure remains limited for several recently approved products, and evidence in pediatric and other underrepresented populations remains incomplete. In severe neurological, neuromuscular, and systemic diseases, manifestations of the underlying disorder and complications of the administration procedure may additionally resemble or amplify toxicity. These considerations mean that the present analysis should be interpreted as a qualitative synthesis of safety patterns and plausible determinants rather than as a quantitative comparison of relative risk or proof of causal mechanisms.

Future development should therefore combine improved molecular design with equally rigorous characterization of delivery, biodistribution, and long-term exposure. Advances in nucleotide and backbone chemistry, sequence optimization, and delivery technologies have already contributed to greater stability, target selectivity, and clinical tolerability (Roberts et al., 2020; Hou et al., 2021; Ruchi et al., 2025). Computational and AI-based approaches may further assist sequence selection, prediction of unintended interactions, and identification of potential toxicity liabilities, but such approaches should complement rather than replace experimental validation, clinical investigation, and regulatory safety assessment. Long-term extension studies, systematic post-marketing pharmacovigilance, and product-specific monitoring will remain essential as clinical exposure increases, and RNA therapeutics expand into broader patient populations.

Overall, the evolution of RNA therapeutics demonstrates that improving safety is not simply a matter of selecting an RNA modality, but of optimizing the entire therapeutic architecture—from sequence and chemical modification to delivery, administration, tissue exposure, and clinical application. A mechanistically informed, product-specific approach to safety assessment, coupled with continued long-term surveillance and individualized risk–benefit evaluation, will be central to the development and responsible clinical use of the next-generation of RNA therapeutics.
